# Hybrid learning: a combination of self-supervised and supervised learning for joint MRI reconstruction and denoising in low-field MRI

**DOI:** 10.1088/1361-6560/ae792b

**Published:** 2026-06-25

**Authors:** Haoyang Pei, Nikola Janjušević, Renqing Luo, Ding Xia, Xiang Xu, William Moore, Yao Wang, Hersh Chandarana, Li Feng

**Affiliations:** 1 Bernard and Irene Schwartz Center for Biomedical Imaging, Department of Radiology, New York University Grossman School of Medicine, New York, NY, United States of America; 2 Center for Advanced Imaging Innovation and Research (CAI2R), Department of Radiology, New York University Grossman School of Medicine, New York, NY, United States of America; 3 Department of Electrical and Computer Engineering, NYU Tandon School of Engineering, New York, NY, United States of America; 4 Biomedical Engineering and Imaging Institute and Department of Radiology, Icahn School of Medicine at Mount Sinai, New York, NY, United States of America

**Keywords:** hybrid learning, deep learning, self-supervised learning, denoising, reconstruction, low-field, lung MRI

## Abstract

*Objective.* Deep learning has demonstrated strong potential for magnetic resonance imaging (MRI) reconstruction. However, conventional supervised learning requires high-quality, high-signal-to-noise-ratio (SNR) reference data for network training, which are often difficult or impossible to obtain, particularly in low-field MRI. Self-supervised learning (SSL) eliminates the need for reference training data but may suffer from degraded performance under low-SNR conditions. To address these limitations, we propose hybrid learning, a new training framework that integrates self-supervised and supervised learning for joint MRI reconstruction and denoising when only low-SNR training data are available. *Approach.* Hybrid learning is implemented in two sequential stages. In the first stage, SSL is applied to fully sampled low-SNR data to generate higher-quality pseudo-references. In the second stage, these pseudo-references are then used as targets for supervised learning to reconstruct and denoise undersampled, noisy data. The proposed method was evaluated in four experiments using simulated and real noisy MRI data of the breast, lung, and brain across different field strengths (0.3 T to 3 T), sampling trajectories (Cartesian, spiral, and radial), noise levels, and undersampling ratios. *Main Results.* Hybrid learning consistently improved reconstruction quality relative to both supervised and self-supervised baselines under different acceleration rates, noise levels, and sampling patterns in all experiments. Compared with standard supervised learning using noisy references, it achieved up to 167.70% higher structural similarity index measure (SSIM), 95.41% lower normalized mean squared error (NMSE), and 90.70% lower high-frequency error norm (HFEN). Compared with standard SSL, it achieved up to 23.88% higher SSIM, 60.85% lower NMSE, and 49.13% lower HFEN. *Significance.* Hybrid learning enables improved MRI reconstruction under low-SNR imaging conditions by jointly addressing noise and undersampling. It provides a practical solution for robust deep learning-based reconstruction and is particularly well suited for applications such as low-field MRI, where image quality is limited by reduced SNR.

## 1. Introduction

Rapid magnetic resonance imaging (MRI) techniques play an essential role in clinical imaging by enabling faster scans without compromising diagnostic quality. Traditional acceleration methods, such as parallel imaging, compressed sensing, and their variants, have demonstrated significant value across a wide range of clinical applications (Baert 2007, Chandarana *et al* 2013, Jaspan *et al* 2015, Feng *et al* 2016, 2017, 2020, Feng 2023a, 2023b, Chen *et al* 2024, Feng and Chandarana 2025). More recently, deep learning-based MRI reconstruction methods have shown remarkable promise for fast MRI, enabling higher acceleration rates and improved image quality that were previously difficult to achieve (Aggarwal *et al* 2019, Qin *et al* 2019, Sriram *et al* 2020a, 2020b, Liu *et al* 2019a, 2019b, 2021, 2023, Yaman *et al* 2020, 2021, 2022, Chen *et al* 2021, Zalbagi Darestani and Heckel 2021, Chung and Ye 2022, Hammernik *et al* 2023, Feng *et al* 2024, 2025, Gu *et al* 2024, Zhang *et al* 2024, Vornehm *et al* 2025, Wang and Davies 2025, Janjusevic *et al* 2025a).

Most state-of-the-art deep learning-based MRI reconstruction techniques rely on supervised learning, which requires fully or sufficiently sampled high-quality reference images for training (Aggarwal *et al* 2019, Qin *et al* 2019, Sriram *et al* 2020a, 2020b, Liu *et al* 2019a, 2019b, Chung and Ye 2022, Vornehm *et al* 2025, Janjusevic *et al* 2025a). However, this requirement poses a major challenge under low signal-to-noise ratio (SNR) imaging conditions, where acquiring high-quality reference data is difficult or impossible. This limitation is particularly pronounced in low-field MRI (e.g. <1 T), where reduced SNR and longer acquisition times make it challenging to obtain fully sampled, high-SNR datasets (Campbell-washburn *et al* 2019, Janjušević *et al* 2026).

Self-supervised learning (SSL) methods have been developed to alleviate this dependency by enabling MRI reconstruction without requiring fully sampled training (Yaman *et al* 2020, 2021, 2022, Chen *et al* 2021, 2026, Liu *et al* 2021, 2023, Zalbagi Darestani and Heckel 2021, Feng *et al* 2024, 2025, Gu *et al* 2024, Zhang *et al* 2024, Wang and Davies 2025). While these approaches can achieve reconstruction performance comparable to supervised learning under certain conditions, their reconstruction quality often degrades in low-SNR settings, particularly at higher acceleration rates (Yan *et al* 2024). Therefore, both supervised and self-supervised approaches face limitations when reconstructing accelerated MRI data in low-SNR regimes.

To address this challenge, this work proposes a new deep learning-based MRI reconstruction framework, called hybrid learning, for joint denoising and reconstruction in low-SNR applications where only fully sampled low-SNR data are available. Hybrid learning employs a two-stage training strategy that effectively combines the strengths of self-supervised and supervised learning while mitigating their respective limitations (Pei *et al* 2025b). In the first stage, SSL is applied to fully sampled low-SNR data to generate higher-quality pseudo-reference images. In the second stage, these pseudo-references are then used as training targets for supervised learning to reconstruct undersampled low-SNR data. Once training is complete, the second-stage network can be directly applied to reconstruct new undersampled low-SNR datasets.

The proposed method was evaluated for joint MRI reconstruction and denoising using both simulated and real-world noisy data across different organs, sampling patterns, noise levels, and acceleration rates. The overall hypothesis was that by leveraging SSL to generate intermediate high-quality pseudo-references, hybrid learning improves reconstruction performance in low-SNR conditions compared to either standard supervised or SSL alone.

## 2. Methods

In this section, we review the fundamentals of deep learning-based MRI reconstruction, outline the supervised and SSL strategies adopted in this work, and then describe how hybrid learning is derived by combining these two approaches. The proposed hybrid learning framework is subsequently evaluated in different experiments using both simulated noisy data and real low-field data.

### 2.1. MRI reconstruction using unrolled neural networks

MRI reconstruction from undersampled *k*-space data can be formulated as the following optimization problem:

\begin{equation*}\begin{array}{*{20}{c}} {\hat x = {\text{ }}\mathop {{\mathrm{argmin}}}\limits_x \frac{1}{2}\left\| {\sqrt W \left( {Ex - y} \right)} \right\|_2^2 + \lambda R\left( x \right)} \end{array}\end{equation*}

where *y* denotes the acquired multi-coil *k*-space data and *x* is the reconstructed coil-combined image. The encoding operator $E = FC$ incorporates coil sensitivity maps ($C$) and Fourier encoding ($F$). For Cartesian sampling, $F$ includes the undersampling mask. For non-Cartesian sampling, $F$ is implemented using the non-uniform fast Fourier transform (NUFFT) incorporating the underlying sampling trajectory. A density compensation matrix $W$ is also incorporated for non-Cartesian sampling to account for non-uniform *k*-space sampling density (Pipe and Menon 1999), and the encoding operator is written as $E = \sqrt W FC$. Here, $W$ is split into two square-root terms to ensure that the multi-coil encoding operator and its Hermitian transpose are adjoint (Benkert *et al* 2018). A regularization term $\mathcal{R}\left( \cdot \right)$ is included in image reconstruction with a corresponding parameter $\lambda $.

The density compensation matrix is trajectory-specific. In this work, a standard ramp filter was used for radial sampling (Feng *et al* 2014). Specifically, we have ${W_{n,j}} = \sqrt {X_{n,j}^2 + Y_{n,j}^2} ,{\text{ }}$ where ${X_{n,j}} = {\rho _n}{\mathrm{sin}}\left( {{\theta _j}} \right)$ and ${Y_{n,j}} = {\rho _n}{\mathrm{cos}}\left( {{\theta _j}} \right)$, with ${\rho _n}$ denoting the radial coordinate of the $n$-th sample and ${\theta _j}$ denoting the angle of the $j$-th spoke. For spiral sampling, $W$ was computed using a Jacobian-based density compensation derived from the sampled *k*-space trajectory following Hoge *et al* (1997). In all cases, $W$ was computed for each sampling pattern and normalized by its maximum value. For Cartesian sampling, $W$ was not needed and was set as the identity matrix ($W = I$).

The reconstruction problem can be solved using iterative optimization algorithms such as gradient descent. This process can also be implemented using an unrolled network, such as the end-to-end variational network (E2E-VarNet) (Sriram *et al* 2020b), where the regularization can be learned through convolutional neural networks (CNNs) (Ronneberger *et al* 2015), as expressed in equation (2)

\begin{equation*}\begin{array}{*{20}{c}} {{x^{i + 1}} = {\text{ }}{x^i} - {\mu ^i}{C^H}F_{\text{ }}^H\sqrt W \left( {\sqrt W FC{x^i} - \sqrt W y} \right) - {\mathrm{CNN}}\left( {{x^i}} \right)} \end{array}.\end{equation*}

Here, ${\mu ^i}$ is a learnable parameter for the $i{\mathrm{th}}$ unrolled block of the neural network. Following the E2E-VarNet implementation (Sriram *et al* 2020b), the corresponding update in multi-coil image space can be written as:

\begin{equation*}\begin{array}{*{20}{c}} {x_{mc}^{i + 1} = {\text{ }}x_{mc}^i - {\mu ^i}\left( {F_{\text{ }}^HWFx_{mc}^i - x_{mc}^0} \right) - C\left[ {{\mathrm{CNN}}\left( {{C^H}x_{mc}^i} \right)} \right]} \end{array}\end{equation*}

where $x_{{\mathrm{mc}}}^0$ denotes the initial multi-coil undersampled image.

**Figure 1.**
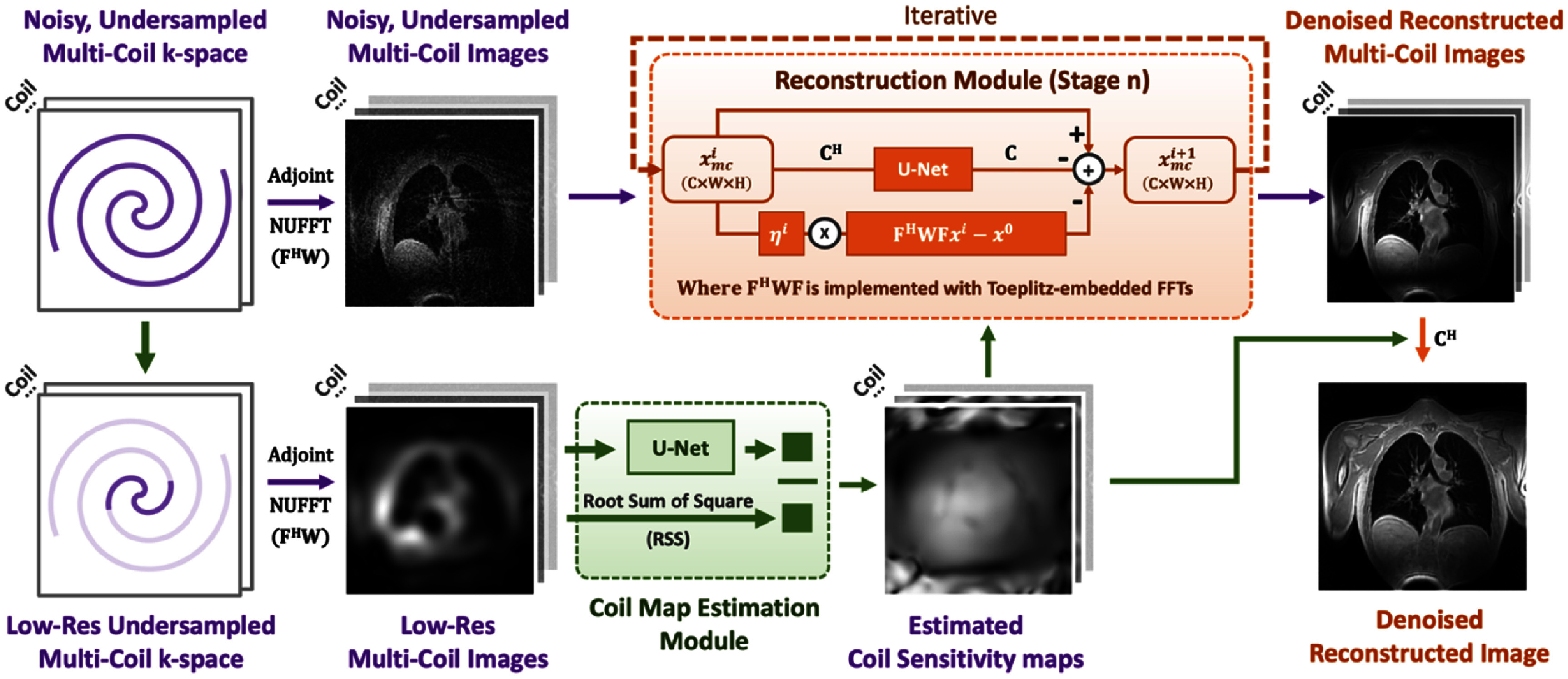
The architecture of the unrolled network for non-Cartesian (spiral) MRI reconstructions (e.g. Breath-Hold Spiral MRI of the Lung at 0.55 T). This unrolled network consists of a reconstruction module and a coil sensitivity estimation module. The reconstruction module employs multiple small U-Nets to model iterative gradient descent updates, where a CNN estimates the gradient of the regularization function. Meanwhile, the coil sensitivity estimation module uses another small U-Net to estimate coil sensitivity maps from the center of the *k*-space data, which are then incorporated into the reconstruction process.

Based on equation (3), an unrolled network architecture can be constructed for both Cartesian and non-Cartesian MRI reconstruction. Figure 1 shows the detailed implementation of the proposed unrolled network for spiral MRI reconstruction, while supporting information figures S1 and S2 provide corresponding implementations for radial and Cartesian sampling, respectively. The reconstruction consists of cascaded unrolled blocks that combine physics-based data consistency (DC) updates with CNN-based regularization modules. In addition, the network incorporates a coil sensitivity estimation module implemented using a U-Net to estimate coil sensitivity maps from the *k*-space center, which has been shown to improve reconstruction performance (Sriram *et al* 2020b).

During reconstruction, the model takes the initial multi-coil image ($x_{mc}^0 = F_{\text{ }}^HWy$) as input. The coil sensitivity maps are first estimated, and the reconstruction module then applies iterative updates to generate the reconstructed multi-coil image $x_{{\mathrm{mc}}}^I$. Finally, the estimated coil sensitivity maps are used to combine $x_{{\mathrm{mc}}}^I$ into the final reconstructed image $\hat x$.

The network implementation used in this work is based on an unrolled architecture derived from gradient descent (Sriram *et al* 2020b), which provides a flexible framework for incorporating physics-based DC and learned regularization. Other unrolled network architectures have also been proposed for MRI reconstruction, including models designed for non-Cartesian sampling such as NC-PDNet (Ramzi *et al* 2022). These architectures can be selected based on specific sampling trajectories and targeted applications.

### 2.2. Supervised and SSL for MRI reconstruction

To train the reconstruction network, appropriate loss functions need to be defined. Depending on how the loss is constructed, deep learning-based MRI reconstruction methods can be broadly categorized into supervised learning and SSL.

In supervised learning, a reconstruction network ${f_\theta }$ is optimized by minimizing the expected difference between images reconstructed from undersampled data $\hat x = {f_\theta }\left( {{F^H}Wy} \right)$ and fully sampled (or sufficiently sampled without noticeable artifacts) high-quality reference images ${x_{{\mathrm{ref}}}}$ using a predefined loss function .

\begin{equation*}\begin{array}{*{20}{c}} {\theta = {\text{ }}\mathop {{\mathrm{argmin}}}\limits_\theta \left( {\mathbb{E}\left[ {{\mathcal{{\mathrm{L}}}_{SL}}\left( {\hat x,{x_{{\mathrm{ref}}}}} \right)} \right]} \right)} \end{array}.\end{equation*}

In practice, this expectation is approximated by averaging the loss across all samples in the training datasets. Once training is complete, the network can be directly applied to new undersampled data for image reconstruction. This approach is widely used and has become the benchmark in many studies (Aggarwal *et al* 2019, Qin *et al* 2019, Sriram *et al* 2020a, 2020b, Liu *et al* 2019a, 2019b, Chung and Ye 2022, Ramzi *et al* 2022, Vornehm *et al* 2025, Janjusevic *et al* 2025a). However, the requirement for high-quality references poses a major challenge under low-SNR imaging conditions, such as in low-field MRI, where high-quality training references are difficult or impractical to acquire.

SSL has emerged as a promising alternative for MRI reconstruction without requiring fully sampled reference images (Yaman *et al* 2020, 2021, 2022, Chen *et al* 2021, Liu *et al* 2021, 2023, Zalbagi Darestani and Heckel 2021, Feng *et al* 2024, 2025, Gu *et al* 2024, Zhang *et al* 2024, Wang and Davies 2025). A widely adopted approach employs a DC loss or a model consistency loss to ensure that reconstructed images remain consistent with the acquired measurements. Additional priors or specifically designed regularizations, such as measurement splitting and equivariant transformations, can be incorporated to further improve reconstruction performance. A representative self-supervised MRI reconstruction method is SSDU (SSL via data undersampling) proposed by Yaman *et al* (2020). In SSDU, each undersampled *k*-space dataset is divided into two disjoint subsets (referred to as Set A and B, or ${y_{\mathrm{A}}}$ and ${y_{\mathrm{B}}}$ hereafter). One set (Set A) is used for network training, while the other set (Set B) enforces DC. The reconstruction network ${f_\theta }$ can be optimized by this self-supervised loss ${\text{ }}{\mathcal{L}_{SSL}}$ as:

\begin{equation*}\begin{array}{*{20}{c}} {\theta = \mathop {{\mathrm{argmin}}}\limits_\theta \left( {\mathbb{E}\left[ {{\text{ }}{\mathcal{L}_{SSL}}\left( {F_{\mathrm{B}}^H{W_{\mathrm{B}}}{F_{\mathrm{B}}}C{f_\theta }\left( {F_{\mathrm{A}}^H{W_A}{y_{\mathrm{A}}}} \right),F_{\mathrm{B}}^H{W_{\mathrm{B}}}{y_{\mathrm{B}}}} \right)} \right]} \right)} \end{array}.\end{equation*}

Here, ${F_{\mathrm{A}}}{\text{ }}$ and ${F_{\mathrm{B}}}{\text{ }}$ represent two FFT/NUFFT operators for Set A and Set B, incorporating corresponding sampling mask/trajectories, respectively. Once trained, the network can be applied to reconstruct new undersampled MRI data without data splitting. In addition to image reconstruction, this training strategy can also provide implicit denoising because noise in Set A and Set B is independent, while the underlying signal remains the same. As a result, the model learns to reconstruct clean images by minimizing the differences between pairs of noisy observations without the need for ground-truth clean images, similar to the Noise2Noise model (Lehtinen *et al* 2018). More recently, the SSDU technique has also been extended to different variants to achieve improved reconstruction performance, as described in the literature (Yaman *et al* 2022, Millard and Chiew 2024, Zhang *et al* 2024, Alçalar and Akçakaya 2025).

In addition to measurement splitting, several other SSL strategies have been proposed. For example, Liu *et al* proposed an SSL approach based on iterative data refinement, which employs multi-stage self-supervised training to progressively improve the quality of the training data and thereby reduce the bias introduced by the self-supervised data setting (Liu *et al* 2023). Chen *et al* developed equivariant imaging (EI), a self-supervised reconstruction method that incorporates both the DC loss and an invariant set consistency loss by leveraging natural signal equivariances (Chen *et al* 2021, Wang and Davies 2025). However, despite these advances, the performance of self-supervised methods can degrade under low-SNR conditions, particularly at higher acceleration rates (Yan *et al* 2024).

### 2.3. Hybrid learning: a combination of self-supervised and supervised learning

**Figure 2.**
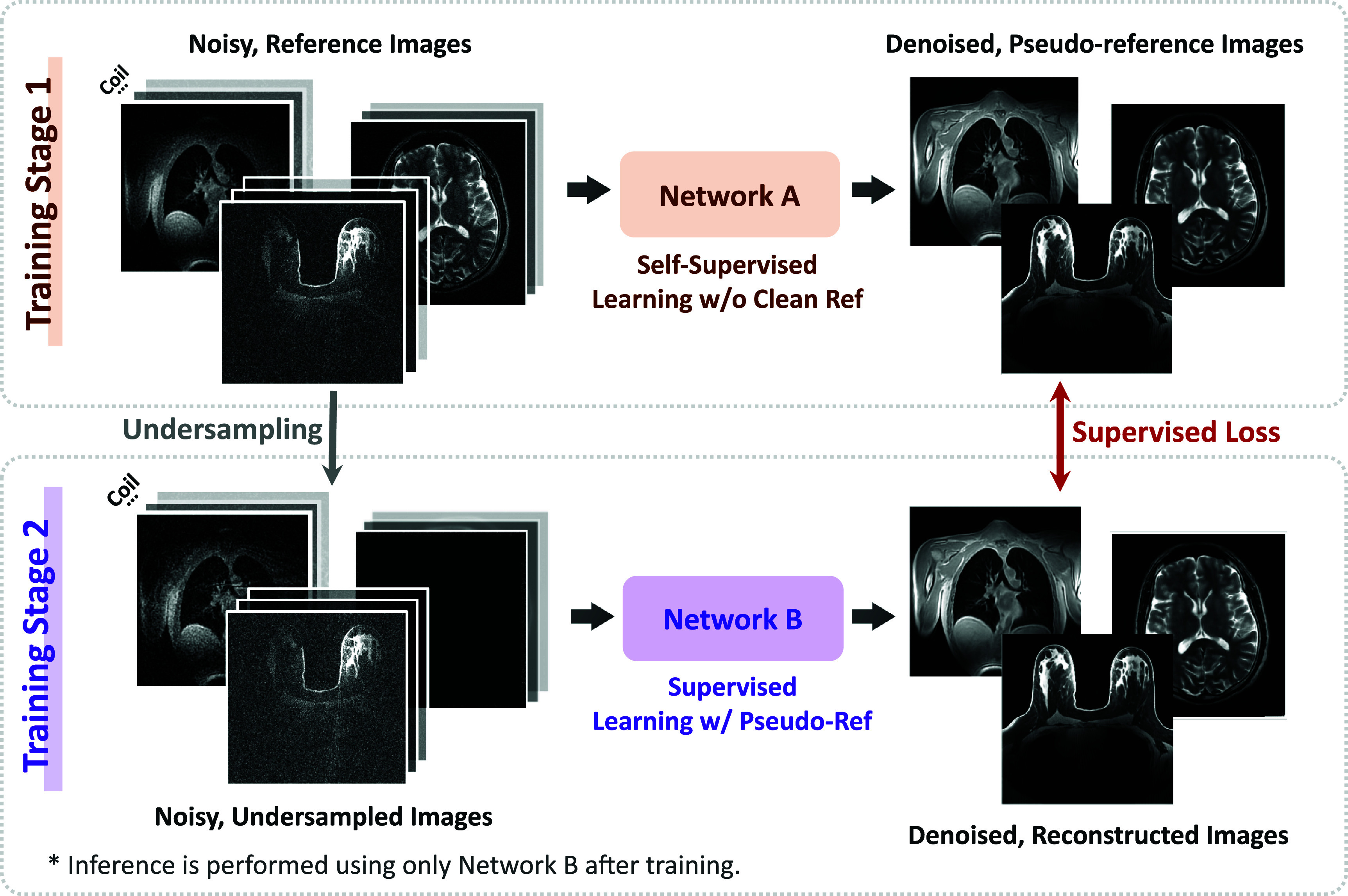
General hybrid learning pipeline. Hybrid learning uses a two-stage framework that combines the strengths of self-supervised and supervised learning. In the first stage, self-supervised learning with Network A is applied to fully sampled low-SNR reference data to generate higher-quality denoised pseudo-reference images. In the second stage, the pseudo-references generated in the first stage are used as training targets for supervised learning with Network B to reconstruct undersampled low-SNR data. After training, the second-stage Network B can be directly applied to reconstruct new undersampled low-SNR datasets. Both Network A and Network B use the same unrolled reconstruction architecture illustrated in figure 1, but are initialized separately and trained using different learning strategies.

To address the limitations of both supervised and SSL, we propose a hybrid learning strategy that combines the two approaches within a two-stage training framework for joint MRI reconstruction and denoising in low-SNR settings, where only fully sampled low-SNR training data are available. Figure 2 provides a conceptual overview of the hybrid learning framework, which consists of two sequential training stages that combine self-supervised and supervised learning for reconstructing accelerated MRI data under low-SNR conditions.

The overall hybrid learning pipeline is as follows. We assume access to a fully sampled, low-SNR *k*-space database with N datasets, denoted as ${D^{\left( 1 \right)}} = \left\{ {y_j^{\left( 1 \right)}} \right\}_{j = 1}^N$. From ${D^{\left( 1 \right)}}$, an undersampled, low-SNR *k*-space database ${D^{\left( 2 \right)}} = \left\{ {y_j^{\left( 2 \right)}} \right\}_{j = 1}^N$, can be generated through retrospective undersampling using predefined acceleration patterns by discarding a subset of *k*-space samples. The goal of hybrid learning is to develop a two-stage reconstruction model that recovers high-quality images from ${y^{\left( 2 \right)}}$ through joint reconstruction and denoising.

In the first training stage, a neural network ${f_\theta }$ (parameterized by $\theta $), referred to hereafter as Network A, is trained using an SSL scheme (equation (5)) on the fully sampled low-SNR data in ${D^{\left( 1 \right)}}$. The objective of this stage is to generate intermediate high-quality pseudo-reference images by reducing noise while preserving anatomical structures. After training, inference is performed on all datasets in ${D^{\left( 1 \right)}}$ to generate a high-quality pseudo-reference for each dataset:

\begin{equation*}\begin{array}{*{20}{c}} {{{\hat x}_{{\mathrm{ref}},{\text{ }}j}} = {f_\theta }\left( {{F^H}Wy_j^{\left( 1 \right)}{\text{ }}} \right),{\text{ }}j = 1, \ldots ,{\text{ }}N.} \end{array}\end{equation*}

In the second training stage, another neural network ${g_\phi }$ (parameterized by $\phi $), referred to hereafter as Network B, is trained on ${D^{\left( 2 \right)}}$ using supervised learning (equation (4)), where the pseudo-references ${\hat x_{{\mathrm{ref}}}}$ generated in the first stage serve as training targets. After training, only Network B is needed for inference to reconstruct high-quality images from new undersampled, low-SNR *k*-space data $y$ (unseen during training)

\begin{equation*}\begin{array}{*{20}{c}} {\hat x = {g_\phi }\left( {{F^H}Wy} \right)} \end{array}.\end{equation*}

**Figure 3.**
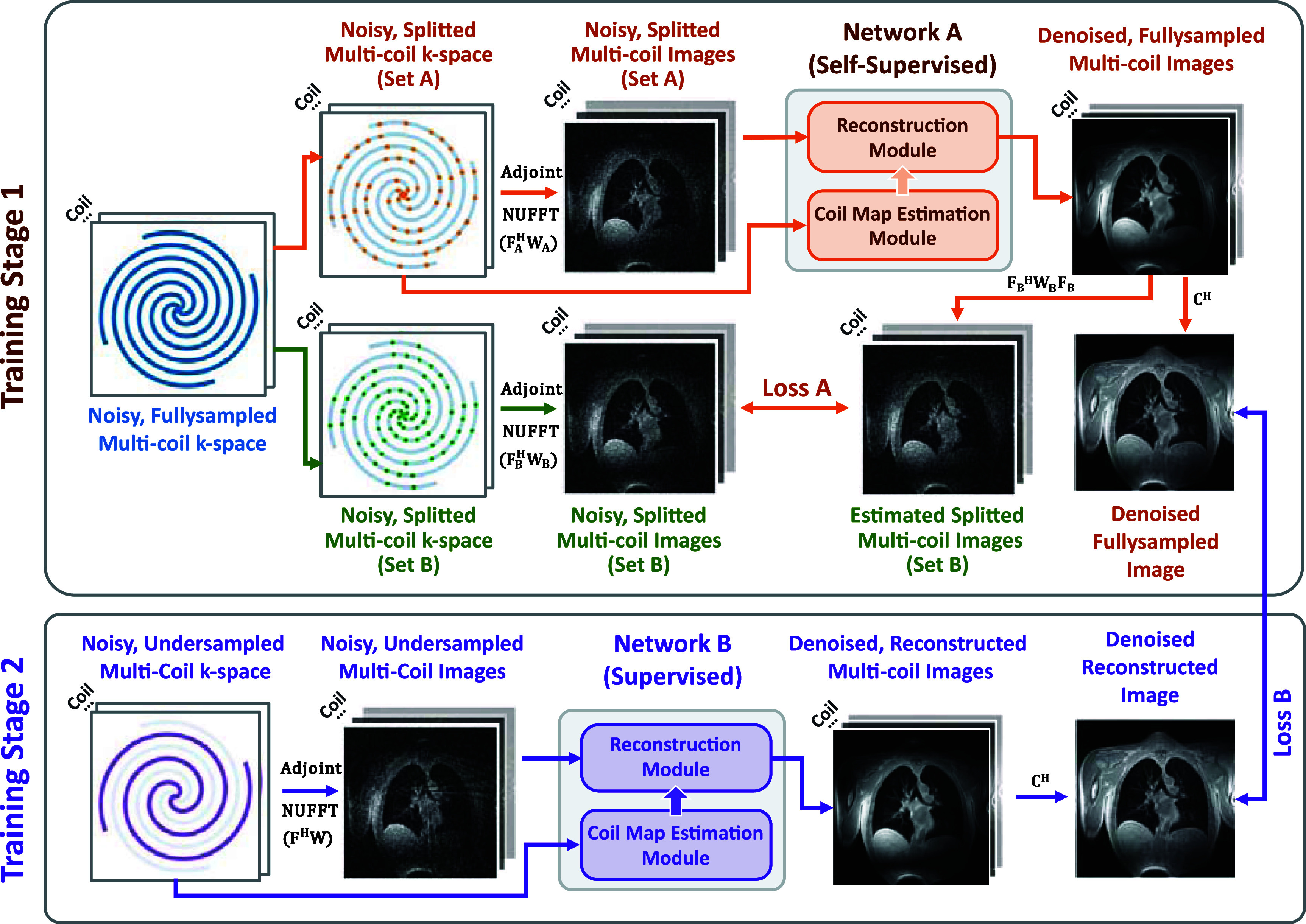
The hybrid learning pipeline for non-Cartesian (spiral) sampling applications (e.g. breath-hold Spiral MRI of the Lung at 0.55 T). In the first stage, Network A is trained for image denoising using fully sampled, low-SNR spiral datasets. Self-supervised learning is used in this step based on an approach adapted from SSDU. After training, inference can be performed directly on fully sampled, low-SNR spiral datasets without *k*-space splitting to generate denoised coil-combined images as high-quality pseudo-references. In the second stage, Network B is trained on the same datasets used in the first stage for joint reconstruction and denoising, using the high-quality pseudo-references as training targets. After training, Network B can be directly applied to new undersampled, low-SNR datasets for joint denoising and reconstruction.

To provide further implementation details, figure 3 shows the hybrid learning pipeline for spiral sampling, while implementations for other sampling trajectories, including radial and Cartesian, follow a similar procedure as shown in supporting information figures S3 and S4, respectively. Both Network A and Network B use the same unrolled reconstruction architecture illustrated in figure 1. Specifically, the reconstruction network consists of 12 cascaded unrolled blocks that combine physics-based DC with CNN-based regularization modules. Coil sensitivity maps are estimated using a separate U-Net-based sensitivity estimation module from the *k*-space center. This reconstruction architecture is used throughout this study, while Network A and Network B differ only in initialization and training strategy for self-supervised and supervised learning, respectively.

In summary, the key rationale behind hybrid learning is that SSL in the first stage improves the quality of the training targets, which then enable more effective supervised learning in the second stage. When the pseudo-references ${\hat x_{{\mathrm{ref}}}}$ closely approximate the true references ${x_{{\mathrm{ref}}}}$, the second stage becomes effectively equivalent to supervised learning with high-quality references. This provides stronger guidance for joint reconstruction and denoising compared with standard supervised learning using noisy references or self-supervised approaches under low-SNR conditions.

### 2.4. Evaluation

The performance of hybrid learning was evaluated in three main experiments and one additional supporting experiment involving both non-Cartesian (radial and spiral) and Cartesian datasets. Experiment 1 was a simulation study on 3 T golden-angle radial breast MRI (Feng *et al* 2014, Kim *et al* 2016, Feng 2022), where synthetic noise was added at different levels to evaluate the performance under different noise conditions. Experiment 2 evaluated hybrid learning using real-world 0.3 T brain MRI datasets with Cartesian sampling, where acquiring fully sampled, high-SNR reference images is time-consuming and SNR is inherently limited. Experiment 3 evaluated hybrid learning on real-world 0.55 T breath-hold spiral lung MRI, where fully sampled, high-SNR reference images are not available. This experiment represents a key clinical target of this study and aims to improve lung MRI at 0.55 T, where reduced field strength improves field homogeneity but also results in inherently lower SNR. Given the absence of high-quality reference images in Experiment 3, an additional supporting experiment using breath-hold spiral lung MRI at 3 T with synthetic noise was included to validate joint reconstruction and denoising in a setting where ground truth images are available. Although lung MRI at 3 T can be affected by field inhomogeneity, it provides high-quality reference images, making it suitable for evaluating reconstruction accuracy. In all experiments, hybrid learning was compared against two baselines: (i) standard supervised learning using noisy references, and (ii) SSL applied directly to undersampled noisy data.

Reconstruction performance was evaluated using the structural similarity index measure (SSIM), normalized mean squared error (NMSE), and high-frequency error norm (HFEN) in all experiments, where HFEN was included to assess high-frequency detail preservation. Statistical significance of quantitative metrics was assessed using a two-sided paired *t*-test to compare performance between methods. The unit of analysis was defined at the subject level (i.e., *n* corresponds to the number of test cases in each experiment). For each subject, metrics were computed for all methods, and paired comparisons were performed across subjects. Because only a small number of pre-specified comparisons were conducted, no multiple-comparison correction was applied.

#### 2.4.1. Experiment 1: simulation study on radial breast MRI at 3 T with synthetic noise

A total of 62 breast MRI datasets from 62 subjects (mean age = 53 ± 14 years) were retrospectively obtained for this simulation study with institutional review board (IRB) approval. All datasets were acquired on a 3 T MRI scanner (MAGNETOM TimTrio, Siemens Healthineers, Erlangen, Germany) without contrast injection using a 3D stack-of-stars golden-angle radial imaging sequence with the following imaging parameters: field of view (FOV) = 320 × 320 mm^3^, matrix size = 320 × 320, slice thickness = 1.1 mm, TR/TE = 4.87/1.8 ms, in-plane spatial resolution = 1 × 1 mm^2^, number of radial views per slice = 288, and number of slices = 102. Total acquisition time was 160 s.

To simulate low-SNR conditions, zero-mean complex Gaussian noise was added to the original radial *k*-space measurements of all datasets at three predefined noise levels (${{{\sigma }}^2} = {\text{ }}0.07,{\text{ }}0.14{\text{ and }}0.21$). Among the 62 datasets, 47 were used for training and validation, and the remaining 15 were used for testing. Three reconstruction methods, including supervised learning with noisy references, SSL, and hybrid learning, were applied to the simulated noisy datasets at four undersampling rates defined by the number of spokes: 200 spokes (scan time = 111 s), 100 spokes (56 s), 50 spokes (28 s), and 30 spokes (17 s). Undersampling was performed by selecting subsets of spokes from the full radial acquisition. The experiment was performed independently for different noise levels.

For quantitative evaluation, self-supervised reconstructions obtained from the original fully sampled 3 T data prior to noise addition were used as reference images. This choice was made because direct gridding reconstruction from 288 spokes still exhibited streaking artifacts that can affect metric computation. These reference images were used only for metric computation and were not used during training.

#### 2.4.2. Experiment 2: evaluation on cartesian brain MRI at 0.3 T

In this experiment, brain MRI datasets from the M4Raw database (Lyu *et al* 2023) were used to evaluate the generalizability of hybrid learning to Cartesian sampling. M4Raw contains real-world Cartesian brain datasets acquired at 0.3 T, with detailed imaging parameters provided in Lyu *et al* (2023).

A total of 128 fully sampled T2-weighted brain datasets were included, with 108 used for training and validation and 20 for testing. Each dataset contained three independent repetitions, which were acquired to improve SNR with signal averaging (Lyu *et al* 2023). All three reconstruction methods were performed on the first repetition of each dataset using fixed 1D uniform undersampling patterns with 14 fully sampled central *k*-space lines at an undersampling rate of 2 (*R* = 1.9 considering fully sampled *k*-space center), 4 (*R* = 3.3), 8 (*R* = 5.1), 12 (*R* = 6.5), and 20 (*R* = 8.5).

To obtain an independent ground truth, a separate self-supervised denoising model (identical to Stage 1 of hybrid learning) was trained using the second and third repetitions. The denoised magnitude images from these two repetitions were averaged to generate reference images for quantitative evaluation. Note that the second and third repetitions were only used to generate reference images, and only the first repetition was used for training the hybrid learning model to ensure independence between training and evaluation.

#### 2.4.3. Experiment 3: evaluation on breath-hold spiral MRI of the lung at 0.55 T

In this experiment, hybrid learning was evaluated on real-world 0.55 T breath-hold spiral lung MRI, where fully sampled high-SNR references were not available. A total of 56 datasets were acquired from 56 subjects (24 males, 32 females; mean age 58 ± 13 years) on a 0.55 T ramped-down MRI scanner (MAGNETOM Aera, Siemens Healthineers, Erlangen, Germany) using a 3D stack-of-spirals ultrashort echo time (3D spiral-UTE) sequence with a uniform spiral sampling trajectory (Mugler *et al* 2015, 2017, Fauveau *et al* 2023). All participants provided written informed consent before MRI scans. Imaging was performed in the coronal orientation with the following parameters: FOV = 500 × 500 mm^2^, matrix size = 256 × 256, in-plane spatial resolution = 1.95 × 1.95 mm^2^, TR/TE = 3.69/0.03 ms, number of slices = 64, flip angle = 5°, and total scan time = 19 s. Each dataset contained 80 spiral interleaves, with a readout duration of 2.2 ms per interleaf. This acquisition was treated as fully sampled (*R* = 1). After reconstruction, images were interpolated to 128 slices with an isotropic voxel size of 1.95 mm^3^.

41 datasets were used for training and validation, while the remaining 15 datasets were used for evaluation. The three reconstruction methods were evaluated at *R* = 2 (40 spiral interleaves, scan time = 9.5 s), *R* = 3 (27 interleaves, 6.3 s), *R* = 4 (20 interleaves, 4.8 s), *R* = 6 (14 interleaves, 3.1 s), and *R* = 8 (10 interleaves, 2.4 s). Because fully sampled high-SNR references were not available at 0.55 T, pseudo-reference images generated from the first stage of hybrid learning were used as surrogate ground truth for computing SSIM, NMSE, and HFEN.

#### 2.4.4. Supporting experiment: simulation study on breath-hold spiral MRI of the lung at 3 T with synthetic noise

In this experiment, a total of 30 lung MRI datasets were acquired on a 3 T MRI scanner (MAGNETOM Prisma, Siemens Healthineers, Erlangen, Germany) from 30 subjects (14 males, 16 females; mean age = 51 ± 16 years). All participants provided written informed consent before MRI scans. Data were acquired during a single breath-hold using the same spiral-UTE sequence as in Experiment 3. MRI scans were performed in the coronal orientation with the following parameters: FOV = 480 × 480 mm^2^, matrix size = 224 × 224, in-plane spatial resolution = 2.1 × 2.1 mm^2^, TR/TE = 2.65/0.05 ms, number of slices = 48, flip angle = 5°, and the total scan time = 18 s. Each dataset comprised 140 spiral interleaves, with a readout duration of 1.16 ms per interleaf. This acquisition scheme produced images free of undersampling artifacts and was treated as fully sampled (*R* = 1). After reconstruction, all images were interpolated to 96 slices with an isotropic voxel size of 2.1 mm^3^.

Similar to Experiment 1, zero-mean complex Gaussian noise was added to the *k*-space measurements of all datasets at three predefined noise levels (${{{\sigma }}^2} = {\text{ }}0.02,{\text{ }}0.04{\text{ and }}0.08$) to simulate low-SNR conditions. 20 datasets were used for training and validation, and the remaining 10 datasets were used for evaluation. Three reconstruction methods were applied to the simulated noisy datasets at *R* = 2 (70 spiral interleaves, scan time = 9 s), *R* = 3 (47 interleaves, 6 s) and *R* = 4 (35 interleaves, 4.5 s). Acceleration was achieved by uniformly discarding spiral interleaves to yield uniform undersampling.

Reconstruction performance was evaluated using SSIM, NMSE, and HFEN, where fully sampled, high-SNR images were used as the ground truth. In addition, fully sampled, denoised images generated from the first stage of hybrid learning (referred to as pseudo-reference hereafter) were also used as a surrogate ground truth to evaluate whether such images could be used for quantitative validation in subsequent real low-field imaging experiments, where fully sampled, high-SNR references are not available.

### 2.5. Implementation

All network weights were optimized using the adaptive gradient descent algorithm optimizer (Kingma and Ba 2014) with a learning rate of 1 × 10^−4^ and a batch size of 1. Each unrolled network consisted of 12 cascaded blocks, corresponding to approximately 8.66 million trainable parameters. Training was performed for 200 epochs in PyTorch (version 2.0) on a server equipped with an NVIDIA Tesla A100 GPU. For supervised learning (including the second stage of hybrid learning), negative SSIM was used for the loss function, as suggested in Sriram *et al* (2020b). For SSL (including the first stage of hybrid learning), a normalized mixed L1–L2 loss was used following Yaman *et al* (2020). All training was performed in a 2D slice-wise manner, where each slice was treated as an independent training sample. Network optimization was conducted directly in the original *k*-space domain (Cartesian, radial, or spiral) consistent with the forward model used in reconstruction.

In self-supervised training (including the first stage of hybrid learning) for lung and breast MRI, a *k*-space splitting ratio between 0.3 and 0.99 was randomly assigned for each spiral interleaf or radial spoke and updated at each backpropagation step. For Cartesian brain MRI, the splitting ratio was set between 0.3 and 0.99 for the first stage of hybrid learning and between 0.4 and 0.6 for standard SSL, as recommended in Yaman *et al* (2020) and confirmed in the experiment. In all cases, a small 4 × 4 central *k*-space region was shared between the two subsets to ensure consistent scaling across sets, as suggested in Janjusevic *et al* (2025b). All splitting settings were separately optimized to ensure fair comparison.

## 3. Results

### 3.1. Results of experiment 1

**Figure 4.**
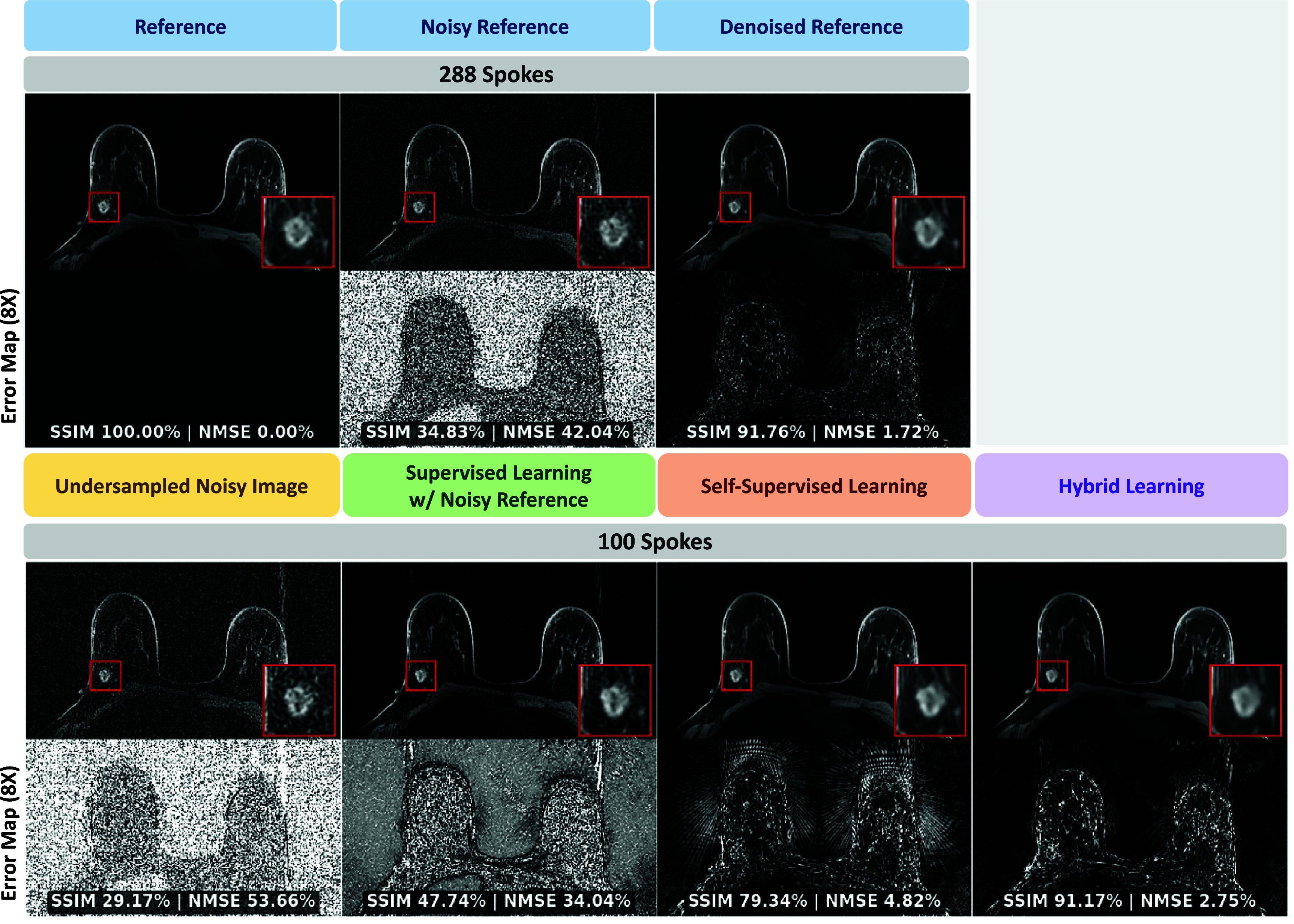
Breast MRI (3 T with simulated noise), radial sampling with 100 spokes, noise level ${\sigma ^2}$ = 0.14. A representative case with a tumor from the simulation study on 3 T breast MRI with radial sampling at 100 spokes and medium-noise level (${\sigma ^2}$ = 0.14), comparing hybrid learning with supervised and self-supervised learning. The 288-spoke reference image (Reference), the 288-spoke noisy image (Noisy Reference), and the 288-spoke denoised pseudo-reference image (Denoised Reference) are included for comparison. Regions of interest (ROIs) and 8× difference maps are provided for reference.

Figure 4 shows a representative case with a tumor from the simulation study on 3 T breast MRI with radial sampling at 100 spokes and medium-noise level (${\sigma ^2} = 0.14$). The 288-spoke reference image, the corresponding noisy image (Noisy Reference), and the denoised image (Denoised Reference) were included for comparison. Both supervised learning (trained with noisy references) and SSL exhibited residual noise and streaking artifacts, as highlighted in the zoomed-in regions. In contrast, hybrid learning achieved improved reconstruction quality, with results that more closely matched the reference images, as reflected visually and in the difference maps.

**Figure 5.**
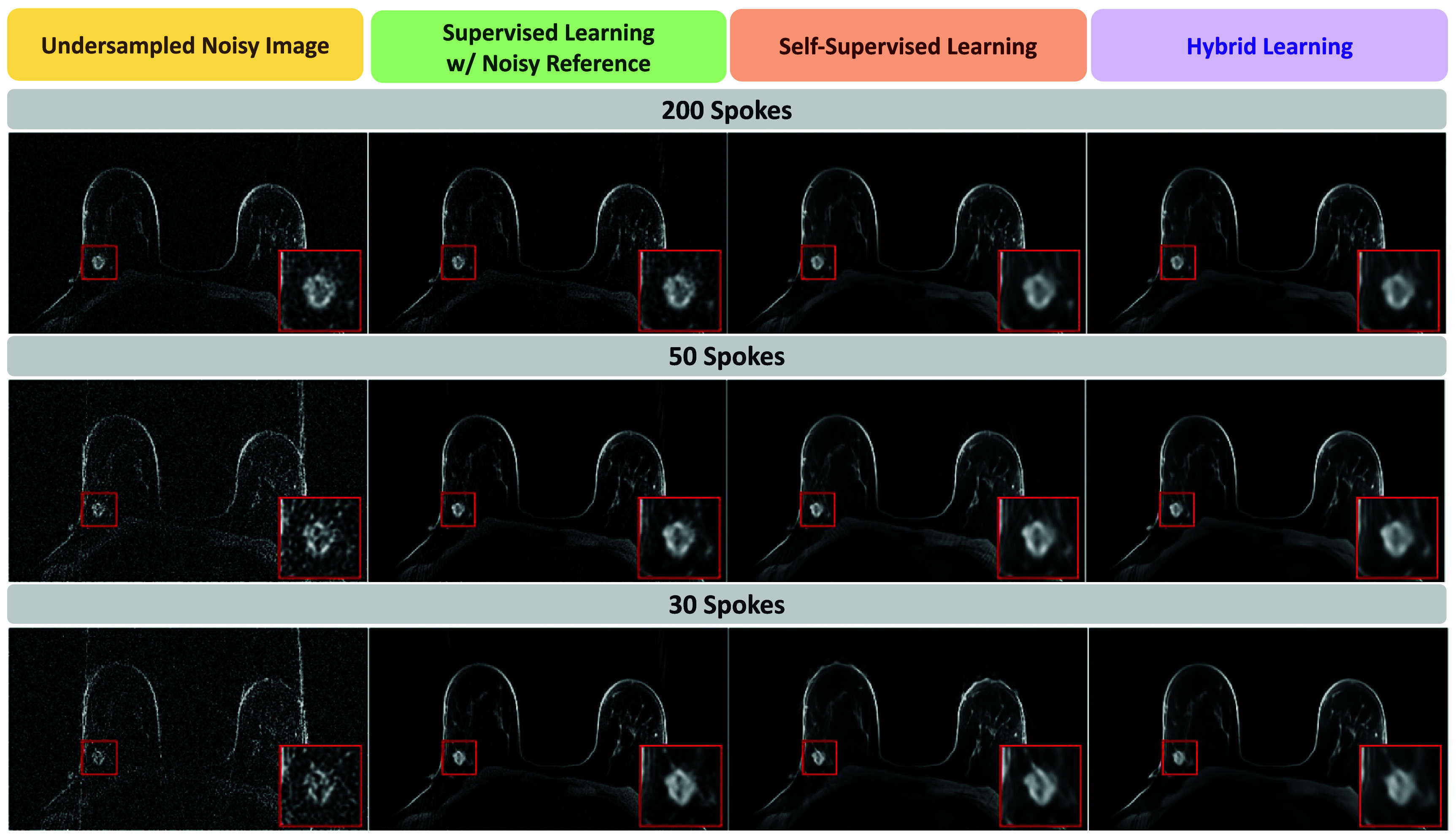
Breast MRI (3 T with simulated noise), radial sampling with 200, 50, and 30 spokes, noise level ${\sigma ^2}$ = 0.14. The corresponding case from figure 4 with 200, 50, and 30 spokes at the medium-noise level, comparing hybrid learning with supervised and self-supervised learning.

Figure 5 presents the same case at other undersampling levels (200, 50, and 30 spokes) under the same noise condition. Hybrid learning consistently outperformed both supervised and SSL across all undersampling rates. In particular, even at 30 spokes, hybrid learning still preserved structural details more effectively. The corresponding difference maps are provided in supporting information figure S8. Supporting information figures S5–S10 further show results from the same case across low, medium, and high noise levels at different undersampling ratios, demonstrating consistent performance improvements of hybrid learning. Supporting information figures S11–S16 show similar trends in an additional case.

Table 1 presents quantitative results averaged over all testing cases (*n* = 15). Hybrid learning achieved higher SSIM and lower NMSE and HFEN than both supervised and SSL methods across all noise levels and undersampling ratios. These improvements were statistically significant (*p* < 0.05), as indicated by the red asterisk (*) and dagger (†), where * denotes comparison with supervised learning using noisy references and † denotes comparison with SSL.

**Table 1.** Breast MRI (3 T with simulated noise), radial sampling at 200, 100, 50, and 30 spokes, noise level 0.07, 0.14, and 0.21. Quantitative results averaged across all testing cases (*n* = 15) at different acceleration rates, comparing hybrid learning with supervised and self-supervised learning. **BOLD** values indicate the best performance for each metric among all compared models.

Metrics	SSIM (%)			NMSE (%)			HFEN (*×*10*^−^*^6^)		
Methods	Supervised learning *w*/Noisy Ref	Self-supervised Learning	Hybrid Learning	Supervised learning *w*/Noisy Ref	Self-supervisedLearning	Hybrid Learning	Supervised learning *w*/Noisy Ref	Self-supervised Learning	Hybrid Learning
=== Low noise level ===									

200 Spokes	53.49 ± 4.66	91.80 ± 1.26	**92.95 ± 1.10** ^*^ ^†^	22.16 ± 2.67	1.77 ± 0.20	**1.64 ± 0.18** ^*^ ^†^	22.80 ± 3.31	4.27 ± 0.56	**3.99 ± 0.45** ^*^ ^†^
100 Spokes	53.53 ± 4.73	85.60 ± 1.89	**91.80 ± 1.19** ^*^ ^†^	23.75 ± 2.93	3.41 ± 0.47	**2.46 ± 0.34** ^*^ ^†^	26.79 ± 4.51	9.36 ± 1.89	**7.68 ± 1.82** ^*^ ^†^
50 Spokes	53.10 ± 4.80	83.52 ± 2.03	**90.44 ± 1.23** ^*^ ^†^	25.43 ± 3.07	4.51 ± 0.54	**3.26 ± 0.36** ^*^ ^†^	31.28 ± 5.01	14.13 ± 2.03	**11.57 ± 1.71** ^*^ ^†^
30 Spokes	55.55 ± 4.40	84.14 ± 1.79	**89.17 ± 1.27** ^*^ ^†^	22.47 ± 2.77	6.00 ± 0.65	**4.34 ± 0.44** ^*^ ^†^	30.66 ± 4.38	24.25 ± 3.41	**17.35 ± 2.20** ^*^ ^†^

=== Medium noise level ===									

200 Spokes	39.44 ± 4.87	88.48 ± 1.67	**90.12 ± 1.55** ^*^ ^†^	46.26 ± 5.06	2.46 ± 0.26	**2.34 ± 0.25** ^*^ ^†^	58.60 ± 10.27	6.60 ± 0.77	**6.24 ± 0.68** ^*^ ^†^
100 Spokes	41.60 ± 4.89	84.30 ± 1.93	**89.19 ± 1.61** ^*^ ^†^	45.48 ± 5.02	4.02 ± 0.48	**3.18 ± 0.36** ^*^ ^†^	58.05 ± 9.81	12.01 ± 1.97	**10.42 ± 1.81** ^*^ ^†^
50 Spokes	42.22 ± 5.05	83.11 ± 1.96	**88.19 ± 1.57** ^*^ ^†^	46.93 ± 5.13	5.06 ± 0.54	**4.10 ± 0.42** ^*^ ^†^	62.47 ± 10.54	17.23 ± 2.16	**15.42 ± 2.03** ^*^ ^†^
30 Spokes	42.42 ± 5.11	83.56 ± 1.31	**86.80 ± 1.63** ^*^ ^†^	46.37 ± 5.20	6.75 ± 0.71	**5.28 ± 0.50** ^*^ ^†^	63.92 ± 10.48	28.66 ± 3.69	**22.19 ± 2.56** ^*^ ^†^

=== High noise level ===									

200 Spokes	32.94 ± 4.93	86.52 ± 1.87	**88.18 ± 1.75** ^*^ ^†^	63.22 ± 6.04	3.03 ± 0.31	**2.90 ± 0.29** ^*^ ^†^	93.66 ± 16.87	8.93 ± 1.03	**8.71 ± 0.98** ^*^ ^†^
100 Spokes	34.52 ± 5.01	83.53 ± 1.97	**87.01 ± 1.72** ^*^ ^†^	62.73 ± 5.98	4.79 ± 0.54	**3.86 ± 0.40** ^*^ ^†^	91.09 ± 16.44	15.62 ± 2.20	**13.75 ± 2.02** ^*^ ^†^
50 Spokes	35.99 ± 5.12	82.38 ± 1.85	**86.02 ± 1.77** ^*^ ^†^	62.67 ± 6.03	5.84 ± 0.62	**4.91 ± 0.48** ^*^ ^†^	92.75 ± 16.53	21.50 ± 2.61	**19.57 ± 2.41** ^*^ ^†^
30 Spokes	36.39 ± 5.13	81.55 ± 1.40	**84.96 ± 1.73** ^*^ ^†^	62.29 ± 6.12	7.80 ± 0.80	**6.26 ± 0.63** ^*^ ^†^	94.01 ± 16.80	33.87 ± 4.32	**27.67 ± 3.40** ^*^ ^†^

^*^ Indicates *p <* 0.05 for Hybrid Learning vs. Supervised learning w/Noisy Ref;

^†^ Indicates *p <* 0.05 for Hybrid Learning vs. Self-supervisedlearning.

### 3.2. Results of experiment 2

**Figure 6.**
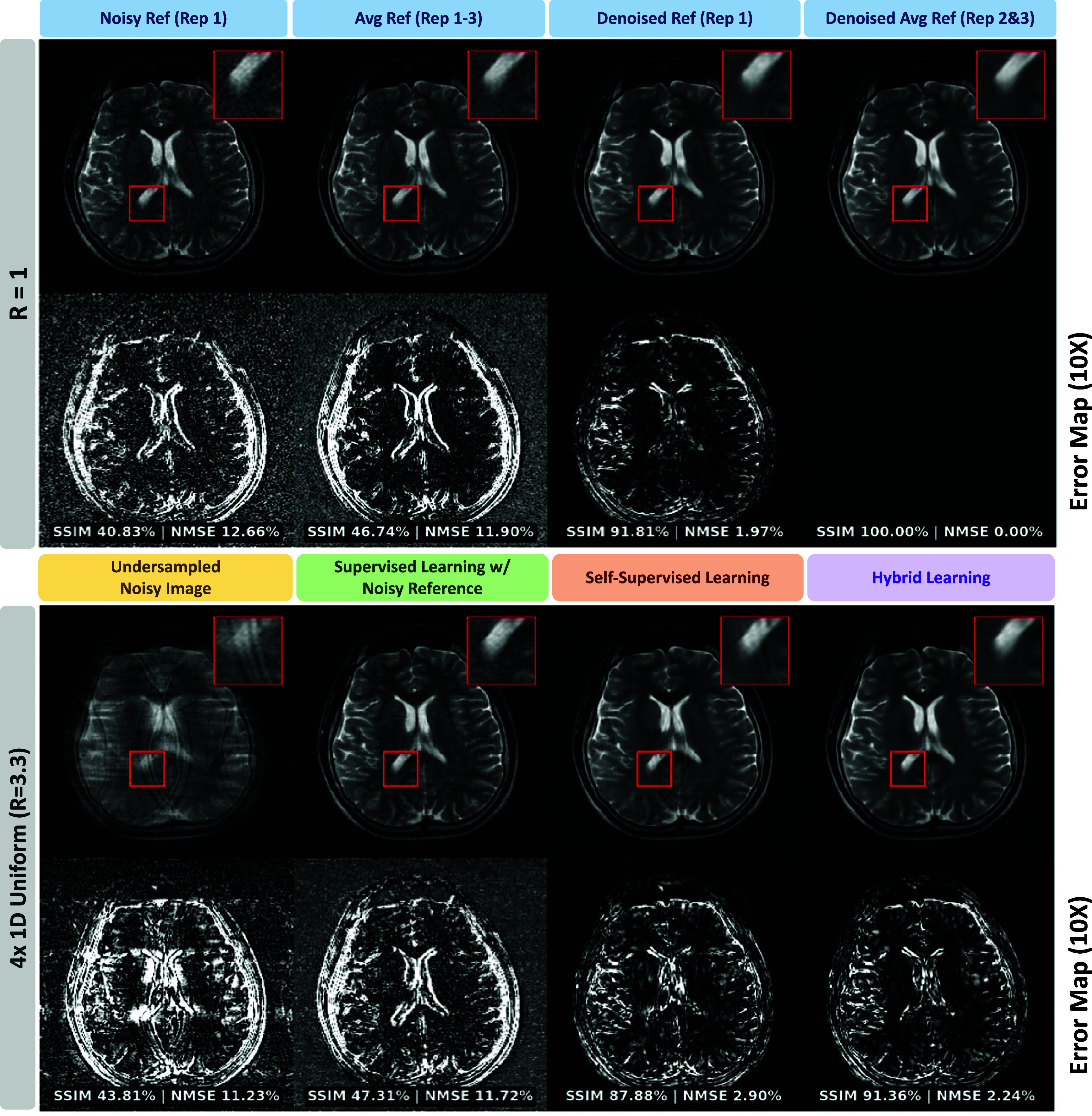
Brain MRI (0.3 T), Cartesian sampling at 4-fold 1D undersampling. A representative case from 0.3 T brain MRI with Cartesian sampling, comparing hybrid learning with supervised and self-supervised learning at 4× 1D uniform undersampling (*R* = 3.3). Top row: (i) Noisy Ref (Rep 1)—the original noisy image from the first repetition, (ii) Avg Ref (Rep 1-3)—the averaged image across all three repetitions, (iii) Denoised Ref (Rep 1)—the denoised image from the first repetition, and (iv) Denoised Avg Ref (Rep 2&3)—the denoised averaged image from the second and third repetitions. Regions of interest (ROIs) and 10× difference maps are provided for reference.

Figure 6 shows a representative case from 0.3 T brain MRI with Cartesian sampling at 4× 1D undersampling (*R* = 3.3). The top row shows fully sampled images under different conditions, including: (i) the original noisy image from the first repetition, (ii) the averaged image across all three repetitions, (iii) the denoised image from the first repetition, and (iv) the denoised averaged image from the second and third repetitions. Reconstructions from both supervised and SSL on the first repetition failed to fully suppress residual noise. In contrast, hybrid learning provided clearer anatomical detail and more effective suppression of noise and artifacts, as highlighted in the zoomed-in regions (red boxes) and difference maps.

**Figure 7.**
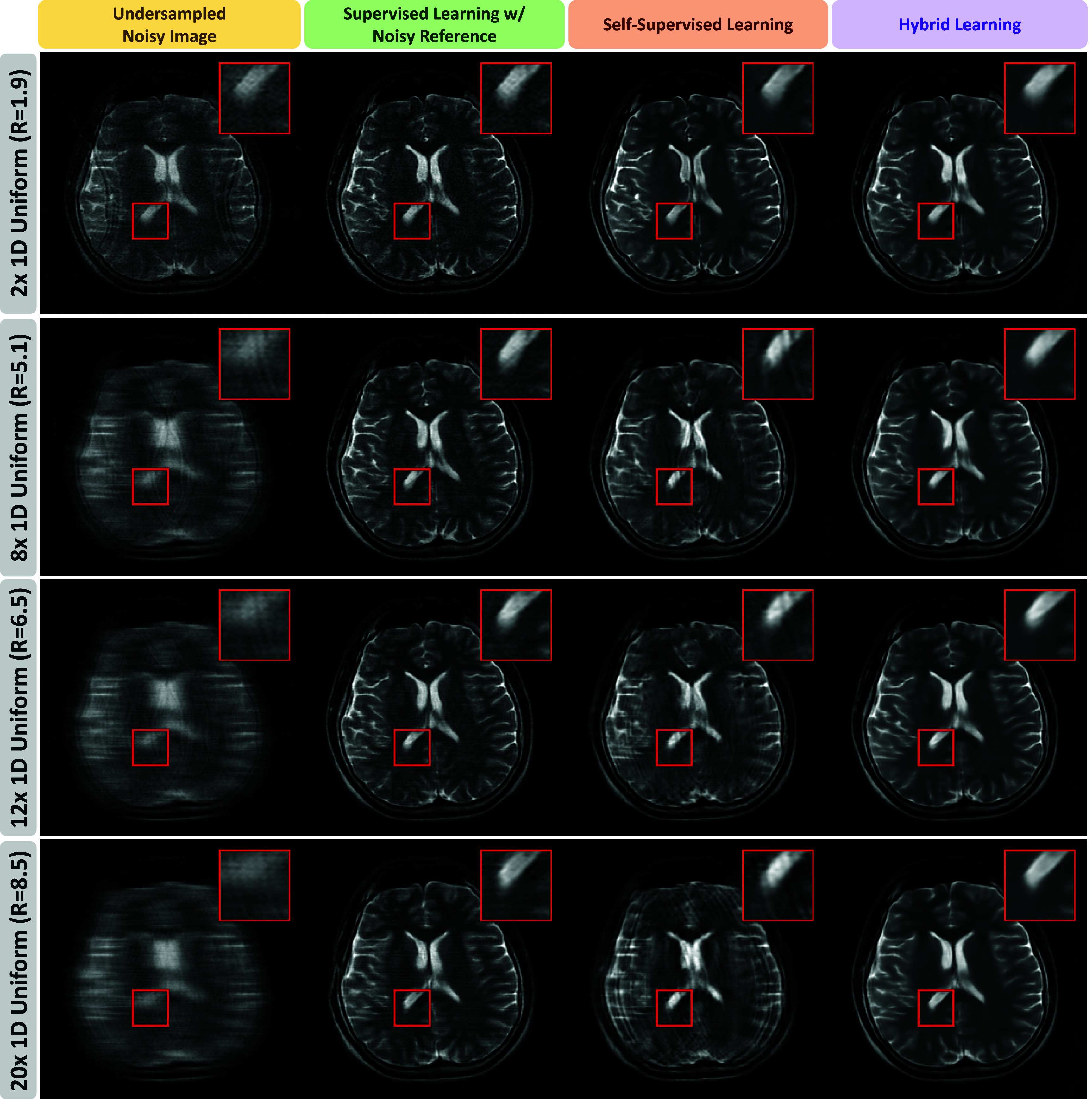
Brain MRI (0.3 T), Cartesian sampling at 2-fold, 8-fold, 12-fold, and 20-fold 1D undersampling. The corresponding case from figure 6 at other acceleration rates (2×, 8×, 12×, 20× 1D uniform), comparing hybrid learning with supervised and self-supervised learning.

Figure 7 presents the same case at other acceleration rates (2×, 8×, 12×, 20× 1D uniform). Hybrid learning continued to outperform both supervised and SSL at these acceleration rates. The corresponding difference maps are shown in supporting information figure S17.

Supporting information figure S18 shows another representative case at a 4× 1D uniform undersampling. Similar trends were observed, with hybrid learning achieving improved image quality and reduced artifacts. Additional results at other acceleration rates are provided in supporting information figure S19.

Table 2**(a)** summarizes quantitative results averaged across all testing cases (*n* = 20). Hybrid learning achieved higher SSIM and lower NMSE and HFEN compared with both reference methods across all acceleration ratios. All improvements were statistically significant (*p* < 0.05), as indicated by the red asterisk and dagger.

**Table 2.** (a) Brain MRI (0.3 T), Cartesian sampling at 2-fold, 4-fold, 8-fold, 12-fold, and 20-fold 1D undersampling. The quantitative comparison of different reconstruction methods across all testing cases (*n* = 20) at 2×, 4×, 8×, 12×, and 20× 1D uniform undersampling. (b) Lung MRI (0.55 T), spiral sampling at *R* = 2, 3, 4, 6, and 8. Quantitative results across all testing cases (*n* = 15) at *R* = 2, 3, 4, 6, and 8. **BOLD** values indicate the best performance for each metric among all compared models.

Metrics	SSIM (%)			NMSE (%)			HFEN (*×*10*^−^*^10^)		
Methods	Supervised learning w/Noisy Ref	Self-supervised Learning	Hybrid Learning	Supervised learning w/Noisy Ref	Self-supervised Learning	Hybrid Learning	Supervised learning w/Noisy Ref	Self-supervised Learning	Hybrid Learning
**(a) Experiment 2: evaluation on cartesian MRI of the brain at 0.3 T**									

2 *×* 1D Uniform	61.14 ± 2.93	88.78 ± 2.84	**93.10 ± 2.80** ^*^ ^†^	11.39 ± 1.36	3.05 ± 1.01	**1.92 ± 0.95** ^*^ ^†^	69.85 ± 12.02	23.17 ± 9.91	**15.71 ± 10.12** ^*^ ^†^
4 *×* 1D Uniform	64.68 ± 2.91	87.86 ± 2.51	**91.54 ± 2.72** ^*^ ^†^	11.10 ± 1.31	3.10 ± 0.84	**2.45 ± 0.90** ^*^ ^†^	79.23 ± 11.98	29.84 ± 9.13	**23.49 ± 10.00** ^*^ ^†^
8 *×* 1D Uniform	66.83 ± 2.97	83.88 ± 2.51	**89.16 ± 2.55** ^*^ ^†^	10.89 ± 1.30	4.00 ± 0.78	**3.35 ± 0.86** ^*^ ^†^	85.17 ± 11.55	39.36 ± 8.62	**36.27 ± 9.91** ^*^ ^†^
12 *×* 1D Uniform	67.51 ± 2.71	78.54 ± 2.72	**88.84 ± 2.55** ^*^ ^†^	10.88 ± 1.12	4.90 ± 0.62	**3.35 ± 0.80** ^*^ ^†^	91.48 ± 11.10	52.85 ± 7.29	**35.70 ± 9.15** ^*^ ^†^
20 *×* 1D Uniform	68.41 ± 2.70	70.01 ± 3.12	**86.73 ± 2.39** ^*^ ^†^	11.37 ± 0.97	7.94 ± 0.64	**4.32 ± 0.70** ^*^ ^†^	116.90 ± 11.83	99.28 ± 7.32	**50.50 ± 8.65** ^*^ ^†^

**(b) Experiment 3: evaluation on breath-hold spiral MRI of the lung at 0.55 T**									

*R* = 2	78.89 ± 5.27	95.95 ± 2.01	**97.91 ± 0.95** ^*^ ^†^	12.54 ± 3.34	5.29 ± 2.10	**2.14 ± 0.90** ^*^ ^†^	5.25 ± 3.22	1.45 ± 0.88	**1.35 ± 0.80** ^*^
*R* = 3	78.54 ± 4.71	94.36 ± 2.31	**96.80 ± 1.17** ^*^ ^†^	12.66 ± 3.22	6.39 ± 2.28	**2.87 ± 1.00** ^*^ ^†^	6.01 ± 3.34	2.19 ± 1.08	**2.02 ± 0.93** ^*^
*R =* 4	77.80 ± 4.49	91.38 ± 2.43	**95.34 ± 1.49** ^*^ ^†^	13.04 ± 3.22	7.88 ± 2.65	**3.70 ± 1.22** ^*^ ^†^	6.95 ± 3.63	3.73 ± 1.58	**3.11 ± 1.16** ^*^ ^†^
*R* = 6	76.09 ± 4.28	86.45 ± 2.52	**92.97 ± 1.80** ^*^ ^†^	13.84 ± 3.18	10.07 ± 2.95	**5.38 ± 1.61** ^*^ ^†^	8.95 ± 3.89	7.48 ± 2.86	**5.36 ± 1.69** ^*^ ^†^
*R* = 8	74.43 ± 4.21	80.38 ± 2.17	**90.46 ± 2.15** ^*^ ^†^	14.54 ± 3.08	12.47 ± 2.96	**6.79 ± 1.73** ^*^ ^†^	10.90 ± 4.18	11.60 ± 3.22	**8.09 ± 2.21** ^*^ ^†^

^*^ Indicates *p <* 0.05 for Hybrid Learning vs Supervised Learning w/Noisy Ref.

^†^Indicates *p <* 0.05 for Hybrid Learning vs Self-supervised learning.

### 3.3. Results of experiment 3

**Figure 8.**
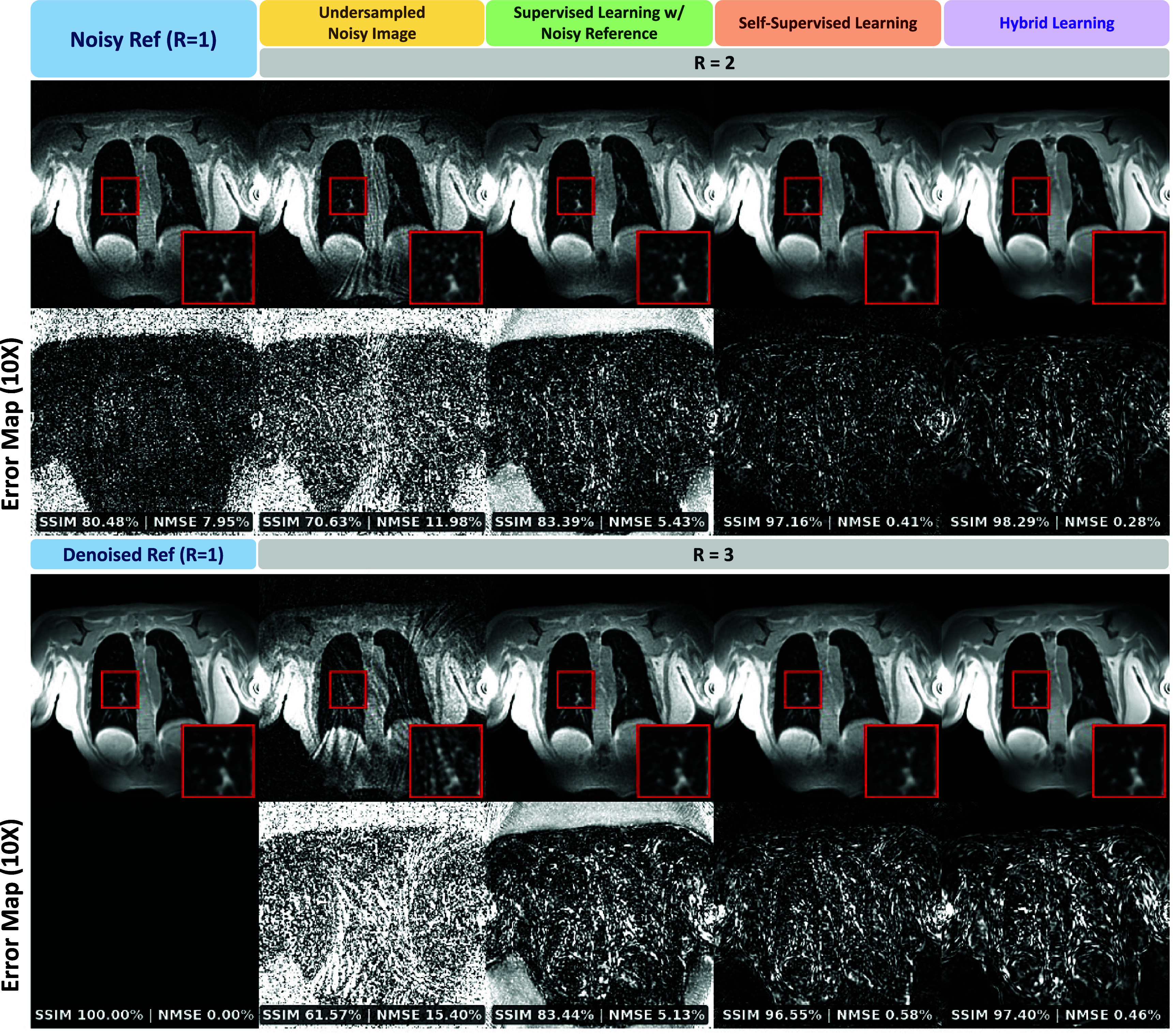
Lung MRI (0.55 T), spiral sampling at *R* = 2 and 3. A representative case from 0.55 T spiral lung MRI at *R* = 2 and *R* = 3, comparing hybrid learning with supervised and self-supervised learning. Regions of interest (ROIs) and 10× difference maps are provided for reference. The fully sampled image (Noisy Reference) shows visible noise, whereas the denoised pseudo-reference image (Denoised Reference) provides improved visual quality. Hybrid learning outperforms both supervised and self-supervised learning at both acceleration rates.

Figure 8 shows a representative case from 0.55 T spiral lung MRI at *R* = 2 and *R* = 3. The fully sampled image (Noisy Reference) exhibited noticeable noise due to low-field imaging, whereas the denoised pseudo-reference image showed improved visual quality. Hybrid learning achieved superior reconstruction quality compared with both supervised and self-supervised methods at both acceleration rates.

**Figure 9.**
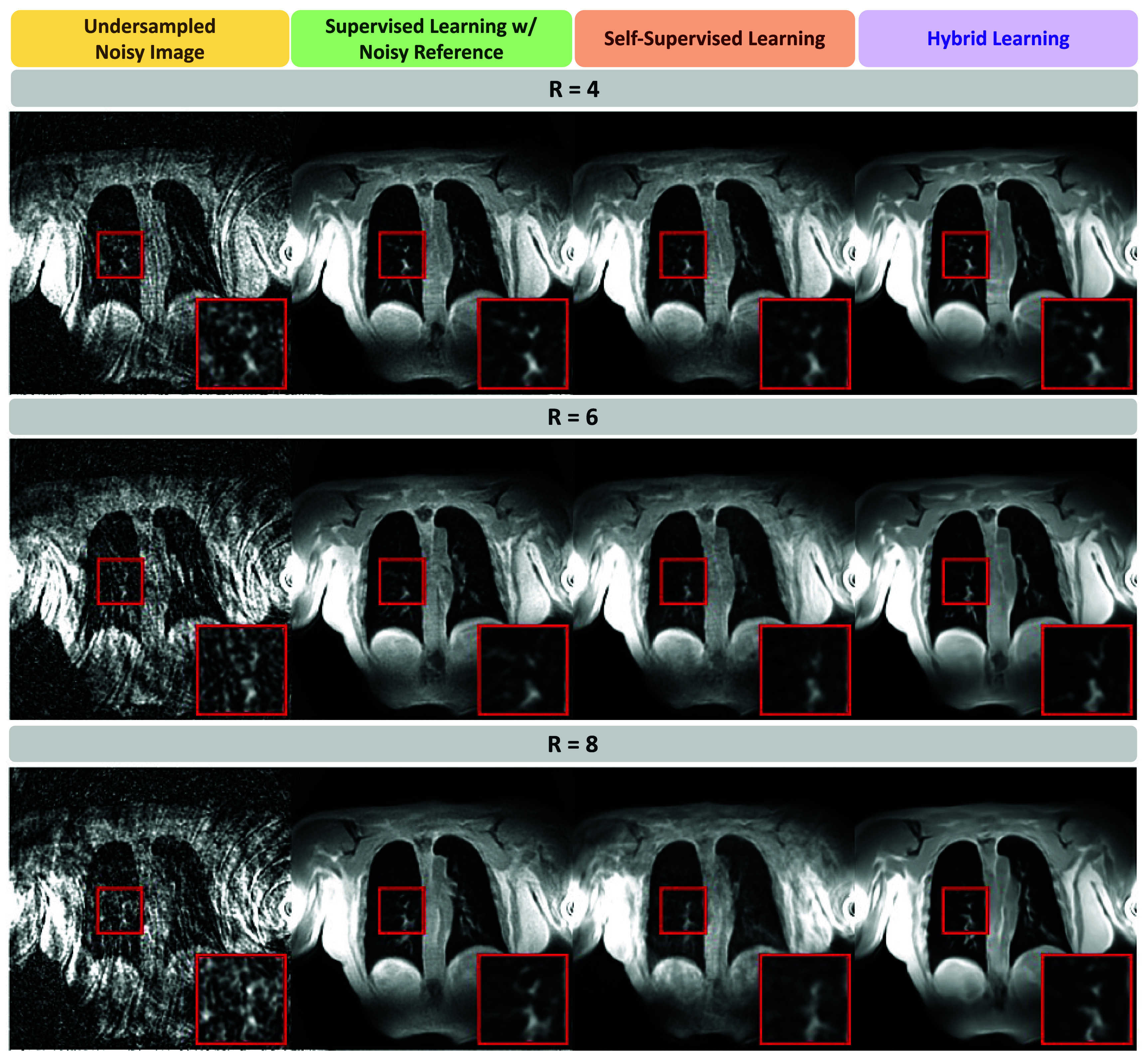
Lung MRI (0.55 T), spiral sampling at *R* = 4, 6, 8. The corresponding case from figure 8 at higher acceleration rates (*R* = 4, 6, and 8), comparing hybrid learning with supervised and self-supervised learning. Hybrid learning consistently provided improved image quality, with better preservation of structural details and fewer artifacts, even as reconstruction became more challenging for all methods at higher acceleration factors.

Figure 9 presents the same case at higher acceleration rates (*R* = 4, 6, and 8). As the acceleration factor increased, reconstruction became more challenging for all methods. Nevertheless, hybrid learning consistently provided improved image quality, with better preservation of structural details and reduced artifacts. Difference maps are provided in supporting information figure S20.

**Figure 10.**
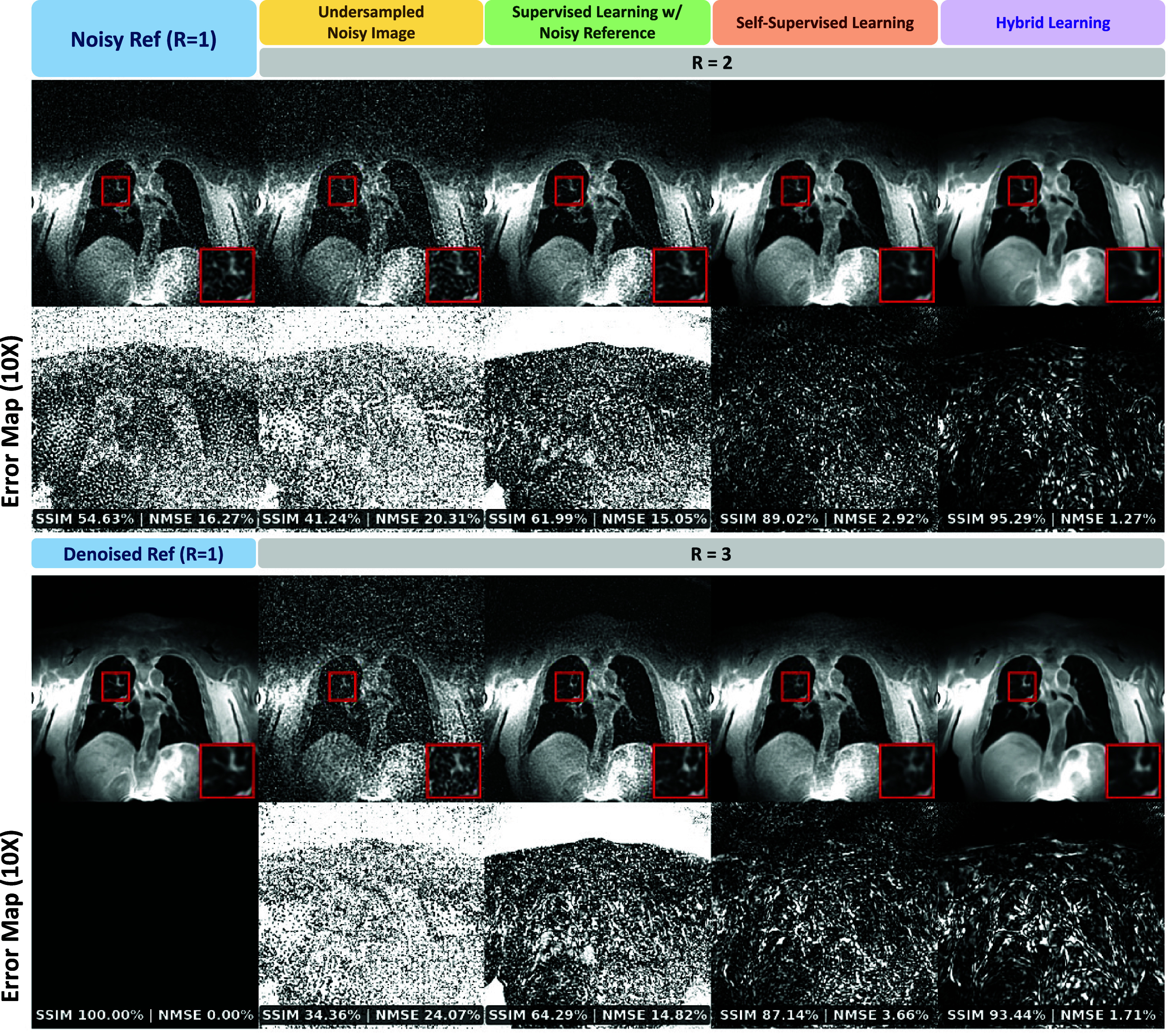
Lung MRI (0.55 T), spiral sampling at *R* = 2 and 3. Another representative case at *R* = 2 and 3 with a higher baseline noise level in the original image (Noisy Ref). Under this more challenging condition, hybrid learning still achieved improved reconstruction quality, with more effective suppression of noise and undersampling artifacts, even though the performance of all methods degraded compared with cases with a lower baseline noise level.

Figure 10 shows another representative case at *R* = 2 and 3 with a lower baseline SNR in the original image (Noisy Ref). Under this more challenging condition, the performance of all methods degraded. However, hybrid learning still achieved improved reconstruction quality with more effective suppression of noise and undersampling artifacts, as highlighted in the zoomed-in regions and difference maps. Supporting information figure S21 shows corresponding results at higher acceleration factors. Supporting information figures S22–S25 present additional cases, where hybrid learning consistently demonstrated improved reconstruction performance.

Table 2(b) summarizes quantitative results across all testing cases (*n* = 15). Hybrid learning achieved higher SSIM and lower NMSE and HFEN compared with both supervised and self-supervised methods at all acceleration factors (*R* = 2–8). All improvements were statistically significant (*p* < 0.05), as indicated by red asterisks and daggers.

The results of the supporting experiment are summarized in a separate Supporting Document (**supporting experiment results**) due to space limitations. Consistent trends were observed compared with the results presented in Experiment 3. These results also support the use of pseudo-references for evaluation and validate the reconstruction performance in low-SNR settings.

## 4. Discussion

In this study, we proposed hybrid learning, a two-stage deep learning framework that integrates SSL in the first stage with supervised learning in the second stage for joint MRI reconstruction and denoising. The proposed training strategy is specifically designed for low-SNR imaging scenarios, where both noise and undersampling degrade image quality and high-SNR reference data are difficult or impractical to obtain. While conventional high-field MRI acquisitions typically provide sufficient SNR for standard learning-based methods, low-field imaging often suffers from substantially reduced SNR, which necessitates more robust reconstruction strategies.

Hybrid learning addresses this challenge by first generating higher-quality pseudo-references from fully sampled low-SNR data using SSL, and then using these pseudo-references to guide supervised training in the second stage. The results demonstrate that hybrid learning consistently outperforms both supervised learning with noisy references and standard SSL when the baseline SNR is low. This is expected, as noisy training references limit the effectiveness of supervised learning, while self-supervised methods alone may degrade under low-SNR conditions.

By improving reconstruction quality under low-SNR conditions, hybrid learning can enable shorter acquisition times while preserving clinically relevant image detail. This is particularly important for low-field MRI and high-resolution imaging, where limited SNR often constrains image quality. One important application is breath-hold lung MRI at 0.55 T, where breath-hold acquisition enables imaging in the inspiratory phase with reduced motion and improved visualization of lung structure, but is constrained by limited scan time and low SNR. Under these conditions, hybrid learning enables joint reconstruction and denoising from undersampled data, which may help reduce breath-hold duration while maintaining diagnostic image quality.

The performance of hybrid learning was evaluated in three main experiments and one supporting experiment. In Experiment 1, a simulation study on 3 T radial breast MRI demonstrated that hybrid learning consistently outperformed both supervised and self-supervised approaches across different noise levels and undersampling conditions. In Experiment 2, the method was evaluated on 0.3 T brain MRI datasets with Cartesian sampling, where independent repetitions enabled reliable quantitative validation. The results confirmed that hybrid learning generalizes across different organs, field strengths, and sampling trajectories.

In Experiment 3, hybrid learning was applied to real-world 0.55 T spiral lung MRI, which represents a clinically relevant low-SNR scenario where high-SNR reference images are not available. Hybrid learning effectively suppressed both noise and undersampling artifacts and maintained image quality across a wide range of acceleration factors. Because fully sampled high-SNR ground truth was not available at 0.55 T, quantitative evaluation was performed using fully sampled denoised images as pseudo-references. To address this limitation, a supporting experiment was conducted on 3 T spiral lung MRI with synthetic noise, where high-SNR reference images were available. This experiment provided a controlled setting to validate reconstruction accuracy and to assess the reliability of pseudo-references. The results demonstrated that pseudo-references generated by hybrid learning closely approximate the true references and can serve as reliable surrogates for both evaluation and training in low-SNR scenarios.

Hybrid learning is not restricted to a specific self-supervised strategy or network architecture. In this work, we implemented SSDU-type SSL in the first stage, but other approaches, such as EI (Chen *et al* 2021, Wang and Davies 2025) or generative models (Chung and Ye 2022), could be used, either independently or in combination with SSDU. Similarly, alternative network architectures beyond the unrolled network used here could be explored (Liang *et al* 2021, Chung and Ye 2022) depending on the application.

Several prior studies have addressed MRI reconstruction under low-SNR conditions. Desai *et al* proposed Noise2Recon (Desai *et al* 2023), which improves SNR robustness for MRI reconstruction but still relies on access to high-SNR data and synthetic noise augmentation during training. Millard *et al* proposed an extension of SSDU, called Robust SSDU (Millard and Chiew 2024), to improve SSL in low-SNR regimes. While this method improves SSL under noisy conditions, it remains limited by the quality of the input measurements. In contrast, hybrid learning improves the training target itself through an initial denoising stage, thus enabling more effective reconstruction under low-SNR and high-acceleration conditions. More recently, Aali *et al* proposed a pipeline that uses a self-supervised denoiser to generate references for supervised reconstruction (Aali *et al* 2025). While conceptually related, their approach relies on Stein’s unbiased risk estimator (SURE)-based denoising (Bigot *et al* 2017), which requires accurate noise-level estimation and is sensitive to estimation errors (Janjušević *et al* 2026). Moreover, their method was only validated on simulated data, leaving its applicability to real low-field MRI untested. In contrast, hybrid learning incorporates denoising directly into the first step as a joint reconstruction-denoising process without explicit noise estimation and is validated on real low-field datasets acquired with both Cartesian and non-Cartesian sampling.

Several studies (Ramzi *et al* 2022, Gu *et al* 2024) have also explored non-Cartesian MRI reconstruction, which is related to the non-Cartesian MRI reconstruction component of our work. For example, Ramzi *et al* (2022) proposed NC-PDNet, an unrolled primal-dual network for non-Cartesian reconstruction. However, NC-PDNet was not specifically designed or optimized for low-field MRI, where high-SNR reference images are not available. In this work, NC-PDNet was evaluated as an additional baseline using standard supervised training with noisy references. The results (supporting information figure S26) show that, similar to other supervised approaches, NC-PDNet exhibits residual noise under low-SNR conditions, whereas hybrid learning provides improved noise suppression and reconstruction quality. This highlights that performance in low-SNR regimes depends not only on network architecture but also on the training strategy.

While the current implementation of hybrid learning employs a sequential training scheme, it can accommodate alternative training strategies. One potential approach is pre-trained weight initialization, where the weights of the second-stage network could be initialized with the pre-trained weights from the first-stage network. Another alternative is joint training of both training stages, where instead of training the two stages sequentially, a parallel training strategy could be implemented. In this approach, the self-supervised loss from the first stage and the supervised loss from the second stage would be combined to jointly update either two separate networks or a single unified network.

Beyond low-field MRI, hybrid learning may also be applicable to other imaging scenarios where high-quality reference data are difficult to obtain. For example, it can be extended to accelerated quantitative MRI (Wu *et al* 2010, Feng *et al* 2021, Li *et al* 2022, Pei *et al* 2023, 2025a, Pei *et al* 2024, Xu *et al* 2025), where fully sampled references are challenging to acquire due to prolonged scan times. In this situation, hybrid learning could improve reconstruction fidelity and enable higher acceleration while preserving structural detail.

Despite its advantages, hybrid learning has several limitations. A key challenge is potential motion inconsistency between the outputs of the first stage and the inputs of the second stage. Such inconsistencies may lead to blurring if the data are not well aligned. While this was not a major issue in the current study, which focused on breath-hold lung MRI and brain imaging, it may become more significant in free-breathing applications. Future work will explore strategies to address this limitation, including incorporating self-supervised constraints in the second stage to improve robustness to motion.

## 5. Conclusion

This work proposes a hybrid learning framework for joint MRI reconstruction and denoising, with a focus on low-SNR imaging applications. By combining self-supervised and supervised learning within a unified framework, hybrid learning effectively addresses the challenges of noise and undersampling when high-quality reference data are not available. The proposed method demonstrates improved reconstruction performance compared with standard supervised and self-supervised approaches and provides a practical solution for robust MRI reconstruction in low-field and other low-SNR imaging scenarios.

## Data Availability

The data cannot be made publicly available upon publication because they contain sensitive personal information. The data that support the findings of this study are available upon reasonable request from the authors. Supporting Figures available at https://doi.org/10.1088/1361-6560/ae792b/data1. Supporting Experiment available at https://doi.org/10.1088/1361-6560/ae792b/data2.
